# BIoMT-ISeg: Blockchain internet of medical things for intelligent segmentation

**DOI:** 10.3389/fphys.2022.1097204

**Published:** 2023-01-12

**Authors:** Asma Belhadi, Jon-Olav Holland, Anis Yazidi, Gautam Srivastava, Jerry Chun-Wei Lin, Youcef Djenouri

**Affiliations:** ^1^ School of Economics, Innovation and Technology, Kristiania University College, Oslo, Norway; ^2^ Department of Computer Science, OsloMet, Oslo, Norway; ^3^ Brandon University, Brandon, MB, Canada; ^4^ China Medical University, Taichung, Taiwan; ^5^ Lebanese American University, Beirut, Lebanon; ^6^ Westsern Norway University of Applied Sciences, Bergen, Norway; ^7^ NORCE Norwegian Research Centre AS, Oslo, Norway

**Keywords:** blockchain, medical internet of things, deep learning, genetic algorithm, segmentation

## Abstract

In the quest of training complicated medical data for Internet of Medical Things (IoMT) scenarios, this study develops an end-to-end intelligent framework that incorporates ensemble learning, genetic algorithms, blockchain technology, and various U-Net based architectures. Genetic algorithms are used to optimize the hyper-parameters of the used architectures. The training process was also protected with the help of blockchain technology. Finally, an ensemble learning system based on voting mechanism was developed to combine local outputs of various segmentation models into a global output. Our method shows that strong performance in a condensed number of epochs may be achieved with a high learning rate and a small batch size. As a result, we are able to perform better than standard solutions for well-known medical databases. In fact, the proposed solution reaches 95% of intersection over the union, compared to the baseline solutions where they are below 80%. Moreover, with the proposed blockchain strategy, the detected attacks reached 76%.

## 1 Introduction

The Internet of Medical Things (IoMT) is a ground-breaking paradigm that connects smart and connected healthcare with individualized medical devices and healthcare information systems (Cao et al., 2019; Gupta et al., 2022). The need for IoMT is motivated by high cost and limited resources of in-hospital treatment, which was clear particularly during the COVID-19 outbreak that has led to considerable growth in the IoMT industry. The IoMT market is anticipated to develop at a compound yearly growth rate of 23.4% between 2021 and 2026 (Zhao et al., 2022). Medical systems can be less taxed by reducing unnecessary hospital visits thanks to IoMT, which directly connects patients with their doctors. IoMT has been particularly essential during the COVID-19 pandemic because it directly increased social estrangement, markedly reduced healthcare response times, and subsequently saved medical costs. In recent years, image segmentation models have become popular (Kheradmandi and Mehranfar, 2022). Since some of these models are already being employed in real-world applications, segmentation models are used in a range of significant domains both in experimental and production settings (Lan et al., 2020; Fang et al., 2021; Ji et al., 2021). Medical imaging is one of the most renowned industries (You et al., 2022). In fact, Bergen Hospital in Norway has started employing these networks for tumor identification since they have shown to be useful (E-Helse, 2019). The models provide the likelihood that the patient has a tumor, which is utilized as a tool to aid clinicians. The segmentation of medical images in IoMT environments is the focus of this work.

### 1.1 Motivation

Federated learning is a cooperative approach to developing a machine-learning model that protects private user information (Zewe, 2022). Several entities train their own models on their own hardware with their own data, numbering in the thousands. Users then send a centralized server their models, which the server then combines to produce a better model and sends the combined model back to all users. Several IoMT based solutions exploring federated learning have been proposed in recent literature (Hakak et al., 2020; Nguyen et al., 2021; Antunes et al., 2022). However, these solutions suffer from several issues: i) They suffer from hyper-parameter optimization issues, ii) Each model achieves high accuracy in a specific kind of data, and iii) They do not provide a secure system for training the model in a federated learning environment. Motivated by the success of hyper-parameter optimization, ensemble learning, and blockchain technology, this paper develops an end-to-end framework for IoMT applied to medical image segmentation.

### 1.2 Contributions

The main contributions of this paper are listed as follows:

1) We develop an ensemble learning model based on U-Net, UNet++, UNet3+, and NAS-UNet for medical image segmentation. In addition, a voting mechanism is used to fuse the local segmentation to the global one.
2) We develop a hyper-parameter optimization strategy based on genetic algorithm to find the optimal model.
3) We propose a blockchain technology to secure the learning process of the developed model in a federated learning environment.
4) We test the suggested framework on IoMT setting, and evaluate it on four medical datasets. The results reveal the superiority of the proposed framework compared to the baseline solutions.

The remainder of this paper is given as follows. Section 2 gives a short overview of the IoMT solutions. Section 3 provides a necessary background on image segmentation in order to make the article self-contained. Section 4 describes the main components of our proposed framework which we reckon BIoMT-ISeg: Blockchain Medical Internet of Things for Intelligent Segmentation. In Section 5, we provide experimental results to assess the performance of BIoMT-ISeg and compares it with the baseline solutions. In Section 6, we discuss the results and their implication while delineating future research direction. Section 7 concludes the paper.

## 2 Related work

This section examines both the existing solutions for IoMT as well as medical image segmentation. The first part examines and reviews IoMT solutions. The state-of-the-art medical image segmentation solutions are described in the second part.

### 2.1 IoMT

Seliem and Elgazzar (2019) developed a blockchain-based security system for IoMT. The four essential parts of the suggested strategy are a cloud server, network cluster, hospital, and smart medical devices. Each medical facility is equipped with a “bolster,” a potent computer that serves as a gateway and server for nearby smart medical devices. The bolster plays the role of a private and safe barrier. It is used to communicate securely with other blocks that are part of the same blockchain. To train intelligent systems using dispersed and locally stored data for the benefit of all patients, Połap et al. (2020) suggested a federated learning strategy that combined decentralized learning with security based on blockchain technology. The work supported the most recent trends in privacy and security for the Internet of Medical Things. Dai et al. (2020) looked into the blockchain-enabled IoMT, which combines blockchain and IoMT. The authors looked into the advantages that blockchain-enabled IoMT could offer, particularly in the fight against COVID-19. They specifically emphasized the prospects offered by blockchain-enabled IoMT and described an architecture for it. After that, the authors looked at the well-known aspects of COVID-19 such as pandemic tracing, social isolation medical data provenances, and remote healthcare. Garg et al. (2020) created a brand-new authentication key agreement system for the Internet of Things. The authors’ system offered safe key management between personal servers and cloud servers as well as between implantable medical devices and personal servers. Secure access to healthcare data stored on cloud servers is also available to authorized users. The cloud servers’ blockchain is where all healthcare data is kept. To show its resilience against potential attacks, a thorough formal security analysis is undertaken, including security verification using the widely used automated validation of security protocols and application tools. Kumar and Tripathi (2021) suggested a consortium blockchain network with smart contracts. They integrated a cluster node for interplanetary flight systems, where smart contracts are deployed initially to authenticate patients and medical devices. After authentication, the same cluster layer is also recommended as a distributed data storage layer, and data is safely transported through the consortium blockchain. By integrating blockchain technology into existing IoMT systems, Li et al. (2021) suggested a framework for IoMT. The authors provided examples of the possibilities offered by IoMT enabled by blockchain. In order to combat the COVID-19 pandemic, the authors also proposed application cases of blockchain-enabled IoMT, including infectious disease prevention, location sharing, contact tracing, and the supply chain of rejected medications. Seliem and Elgazzar (2019) designed a simple blockchain-based system to protect IoMT. The four essential parts of their suggested strategy are a cloud server, network cluster, hospital, and smart medical devices. Each medical facility is equipped with a “bolster”, a potent computer that serves as a gateway and server for nearby smart medical devices. The bolster plays the role of a private and safe barrier. The bolster is utilized for private communication between blocks inside the same blockchain.

### 2.2 Medical image segmentation

Medical image segmentation has the goal to identify different labels in a given medical image. In the context of deep learning, the aim is to design efficient models in order to learn the segmentation function. The input of the model is a medical image, and the output will be the label of each pixel in that image. Huang Q. et al. (2020) proposed a machine learning method for breast medical image segmentation in order to identify tumors. Medical images are first cropped and pre-processed using bilateral filtering, histogram equalization, and pyramid mean shift filtering to remove noise. Simple linear iterative clustering is then performed for grouping the pixel of images into super-pixels. Features are extracted for each super-pixel, where two labels are created. A tumor label is created if the super-pixel contains a tumor, and a normal label is created otherwise. The kNN classifier is then performed to classify the pixels located in the super-pixels as tumor or normal. Adjacent tumor super-pixels are finally merged to segment the tumor of the new image. Amiri et al. (2020) proposed a two-stage medical image segmentation approach for breast lesion detection using U-Net. The first use of the U-Net model aims to detect the lesions. The second use of the U-Net model aims to segment the detected lesions. Lee et al. (2020) introduced the use of channel attention mechanisms to improve CNN performance for breast cancer segmentation. Inter-dependencies of the channels in image are trained by injecting statistical features of each channel (mean of pixel values) on fully connected layers-based networks. The output of this network with the input images are injected into CNN for segmentation. Wu et al. (2020) proposed a encoder-decoder deep learning model for thyroid nodule segmentation on medical image data. The authors’ model contains: i) dense block structure, where any two layers are connected. Batch normalization is used in order to train this dense block. ii) Atrous spatial pyramid pooling is used for creating contextual multi-scale information of input feature map. iii) Model size optimization for reducing the number of parameters is learned, where a further 1 × 1 convolution operation is computed before each convolution layer. The semantic features are obtained from contextual information, and injected into each layer of the decoder module. The hierarchical feature fusion is also performed to merge feature maps of the blocks in the decoder module. Zeng et al. (2021) proposed a hybrid deep learning architecture for fetal medical image segmentation. A combination of V-Net with an attention mechanism is carried out in order to reach the better accuracy of the segmentation results. To deal with large ranges of batch sizes, global normalization is used instead of batch normalization. A mixed loss function based on dice similarity coefficient is developed in order to minimize the error ratio. Note that the dice similarity coefficient is determined by the intersection over the union of the ground truth, and the output of the network. Xue et al. (2021) addressed three issues related to breast lesion medical image segmentation, which are: in homogeneous intensity distributions inside breast lesion regions, ambiguous boundaries due to similar appearance between lesion and non-lesion regions, and irregular breast lesion shapes. CNN is first used for multi-scale feature maps generation. Each CNN layer is connected to a 1 x 1 convolutional layer with a maxpooling operation for detecting breast lesion boundaries. The features of all CNN layers are concatenated and combined with spatial-wise, channel-wise blocks for learning correlation among the generated feature maps, and predicting the output image. Liu et al. (2021) proposed a hybrid deep learning algorithm for detecting prostate cancer. Feature extraction is performed using the Sobel filter, where the features are injected into a RCNN (Regional Convolution Neural Network) for medical image segmentation. Ouahabi and Taleb-Ahmed (2021) developed an encoder-decoder deep learning model for thyroid segmentation. The author’s model adds a new layer which integrates the merits of dense connectivity, dilated convolutions for extracting relevant features, and deals with varied-size regions, respectively. In an effort to include the hierarchical swin transformer into both encoder and decoder of the conventional U-shaped architecture, Lin et al. (2022) developed the dual swin transformer U-Net (DS-TransUNet) for deep medical image segmentation framework. The authors’ framework gains from self-attention computation of the swin transformer and intended dual-scale encoding, which are successful in simulating non-local interactions and multiscale contexts for enhancing the semantic segmentation quality of various medical images.

### 2.3 Discussion

From this short literature review, there are several flaws in current IoMT frameworks, particularly for medical segmentation based solutions. When dealing with real-world scenarios, the first consideration is data privacy and sensitivity. Security in IoMT based solutions is very limited, where many attacks are detected during the training process. These attacks can negatively affect the training process, and therefore degrade the overall performance of the designed system. The second limitation is that each model succeeds for some types of data, and fails for others. Another limitation is that image segmentation models, particularly those based on U-Net architectures, necessitate the tuning of a large number of hyperparameters. This study looks into an intelligent and end-to-end IoMT framework for image segmentation that is based on blockchain technology for security, ensemble learning for training, and the genetic algorithm for hyper-parameter optimization. The next section is described in details the proposed framework.

## 3 Background on image segmentation

An image is “segmented” or “partitioned” into various groups throughout the image segmentation process. For instance, image segmentation is used to distinguish the speaker from the background in Zoom call functionality that lets you alter your background. This is but one use case for image segmentation in the real world. Face identification, video surveillance, object detection, medical imaging, and other fields can all benefit from image segmentation. These applications use two-dimensional data in some cases and three-dimensional data in others.

There are two types of image segmentation: semantic segmentation, and instance segmentation.

• Semantic segmentation, where the objective is to assign a category to each pixel in an image. Semantic segmentation aims to classify each tree in a forest image into the appropriate category.
• Instance segmentation, which accomplishes the same thing as semantic segmentation but goes a step farther. An instance segmentation of the forest image would then separate the trees into tree 1, tree 2, and so on. Instance segmentation aims to separate items of the same category into a series.

**FIGURE 1:**
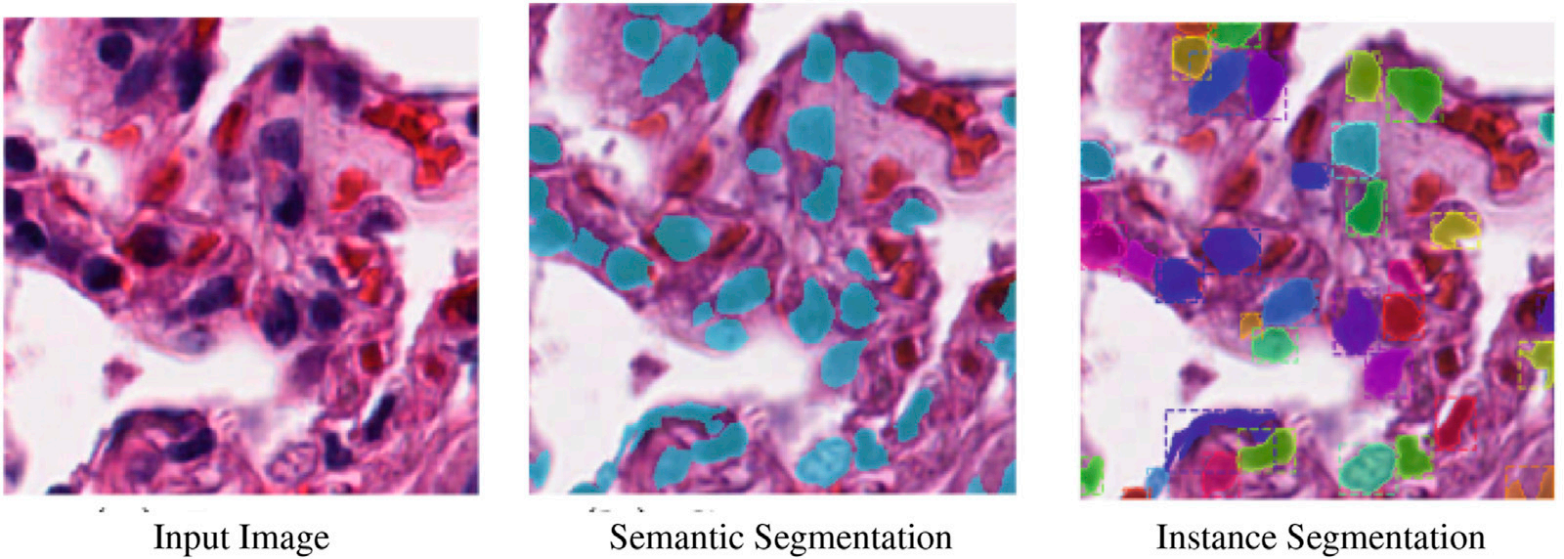
Semantic segmentation vs. instance segmentation (Zhang et al., 2018).

Figure 1 illustrates the distinction between instance and semantic segmentation visually. The terms “semantic segmentation” and “image segmentation” will be used interchangeably. Object segmentation and detection share similarities. Finding various object classes in a given image is the aim of object detection. The object is marked by object detection with a square frame represented by a bounding box. Object detection just displays the location of an object; it does not identify its shape. Object detection does not meet the criterion for several tasks. For instance, the form of the malignant cell is important when estimating the extent of the disease while trying to identify it.

## 4 BIoMT-ISeg: Blockchain medical internet of things for intelligent segmentation

### 4.1 Principle

**FIGURE 2:**
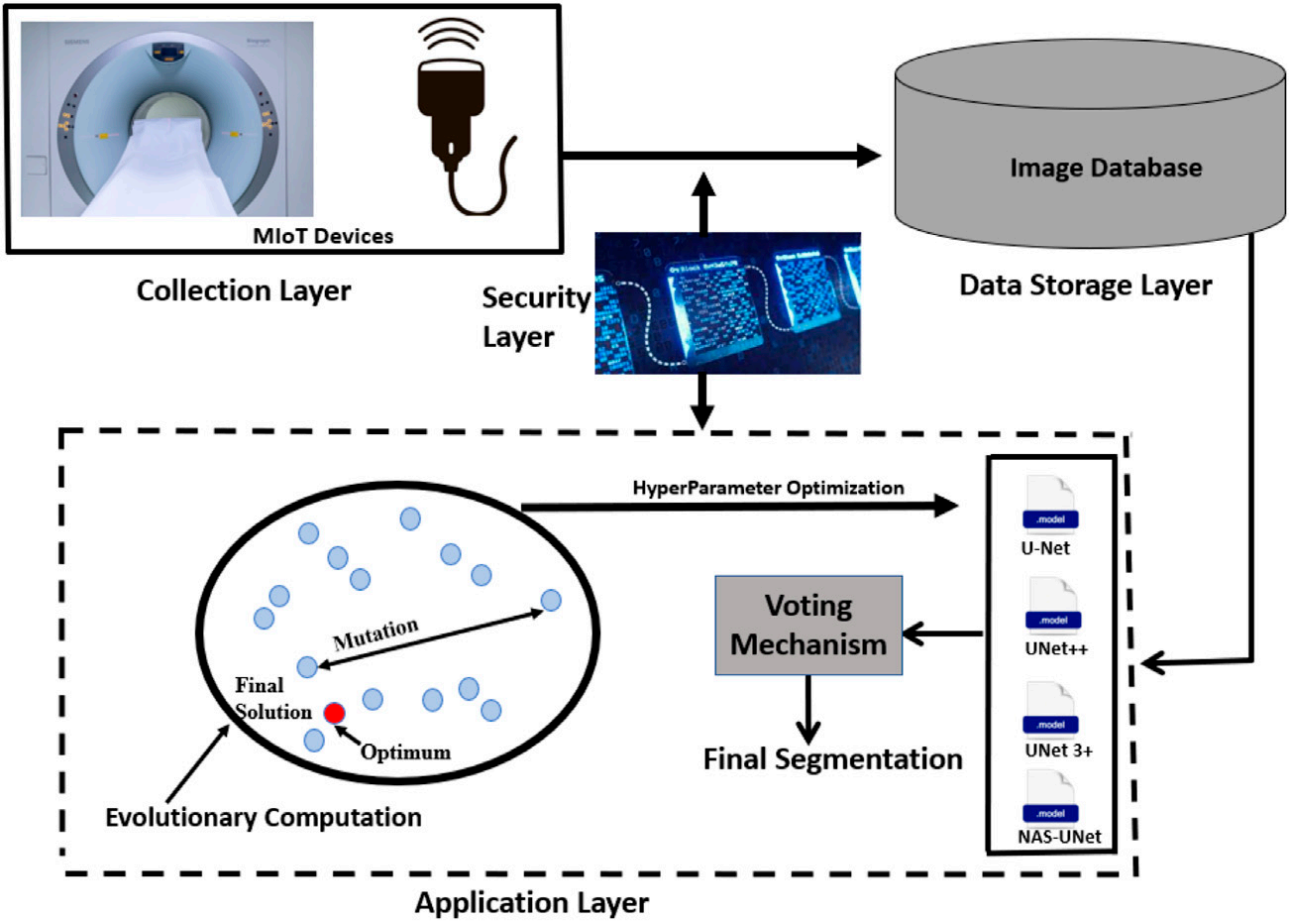
BIoMT-ISeg framework.

Figure 2 shows the BIoMT-ISeg (Blockchain Medical Internet of Things for Intelligent Segmentation) framework. It is made up of four layers: collection, security, data storage, and application. IoMT devices are initially responsible for collecting various types of medical data at the collection layer. Following that, the collected data is communicated to the data storage layer via the security layer implemented by blockchain technology. The IoMT application layer then employs deep learning models to do successful segmentation. It uses several architectures (U-Net, UNet++, UNet 3+, and NAS-UNet) to find the local outputs, while an ensemble learning model is employed to merge local outputs to the global one using a voting mechanism (Sagi and Rokach, 2018). In addition, hyper-parameter optimization is performed using evolutionary computation. This architecture layer is connected to the security layer to secure the training process in a federated learning environment.

### 4.2 Image segmentation

This sub-section presents the deep learning models employed in the developed framework.

#### 4.2.1 U-Net

**FIGURE 3:**
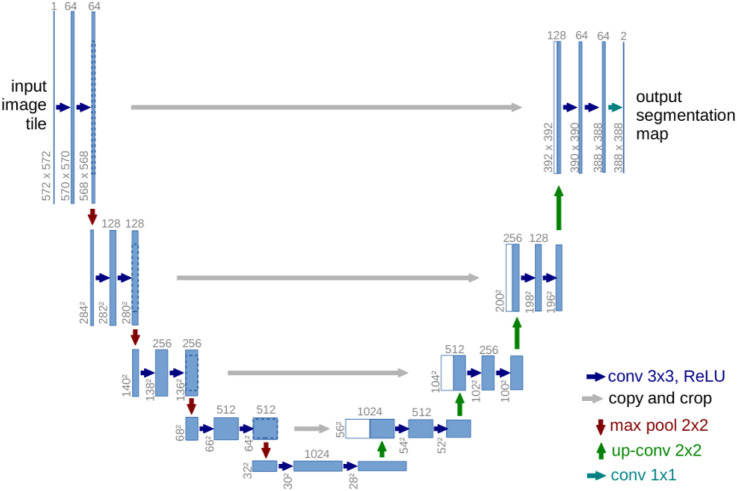
U-Net architecture using long skip connections (Ronneberger et al., 2015).

Ronneberger et al. (2015) suggested U-Net as a medical segmentation technique that needed less annotated samples. Figure 3 illustrates the design, which is based on CNN but uses an encoder-decoder structure with skip connections. Downsampling in the encoder makes use of pooling, whereas upsampling in the decoder substitutes transpose convolution for pooling. The last output layer uses the sigmoid activation function, giving each pixel a value between zero (0) and one (1). The ReLU activation function follows each convolutional layer in both encoder and decoder (Amiri et al., 2020). In their original publication, they test their model on two-dimensional data and obtain cutting-edge outcomes while needing fewer training samples. The authors do state that they are confident their model will be useful for numerous other tasks, though. In fact, U-Net has served as a significant source of inspiration for numerous models created specifically for medical segmentation.

#### 4.2.2 UNet++

UNet++ aims to strengthen some of the flaws and restrictions in the original U-Net for image segmentation (Zhou et al., 2020). Their ultimate objective is to increase image segmentation accuracy. The authors’ conclusions include that the ideal architecture relies on the size and difficulty of the dataset, and that a deeper U-Net network is not always preferable. UNet++ therefore aims to provide a single architecture that produces the best architecture independent of the task at hand. Iterative design is used by the authors to create the UNet++ architecture. They start by developing an architecture called U-Net^e^. U-Net^e^ integrates U-Nets with different depths into a single cohesive structure. In this architecture, each U-Net has its own decoder but shares the encoder to some extent. They also introduce UNet+, which replaces the ensemble’s initial skip connections with connections between each pair of nearby nodes. Each node represents a convolutional block, which is made up of batch normalization, upsampling, and downsampling. Last but not least, the authors introduce dense connections in UNet+, building on the success of DenseNet (Huang et al., 2016). This alteration results in their ultimate architecture, UNet++. Because of this, UNet++ is more adaptable than U-Net and outperforms it in terms of 2D and 3D segmentation. Comparatively speaking, UNet++ does have more parameters than U-Net while U-Net needs 7.8 M, and UNet++ needs 9.0 M.

#### 4.2.3 UNet 3+

**FIGURE 4:**
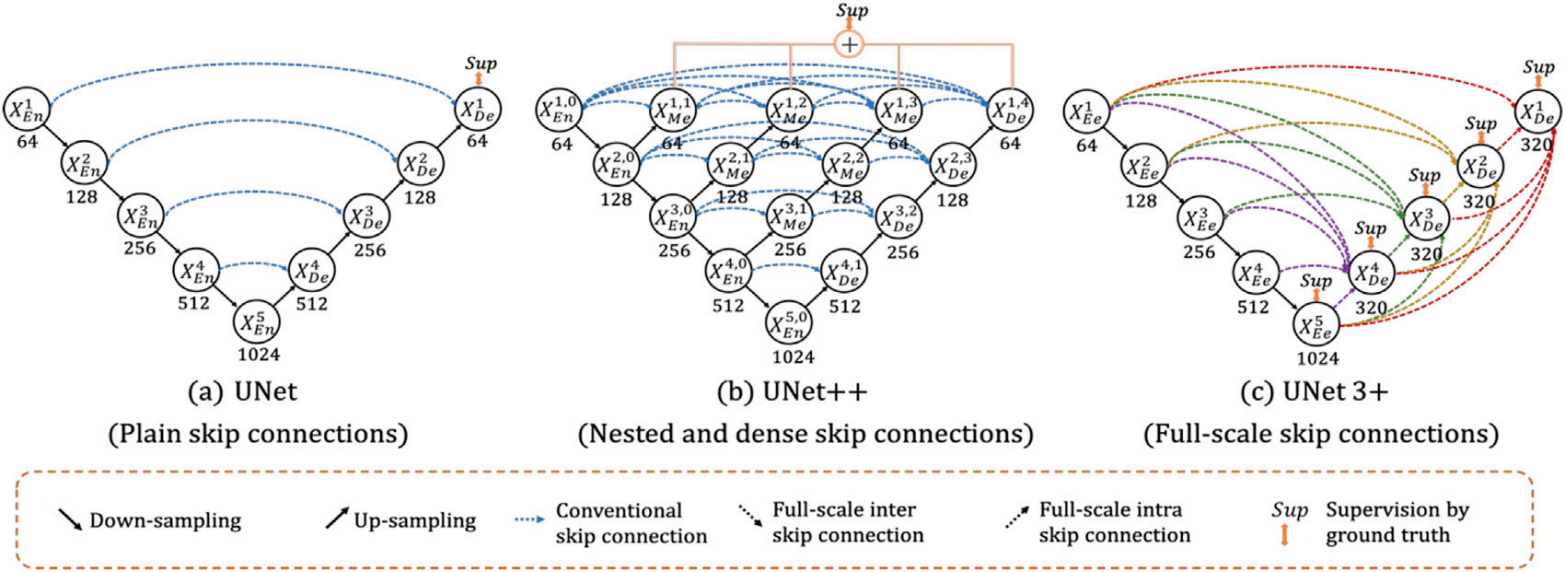
**(A)** U-Net, **(B)** UNet++, and **(C)** UNet 3 + comparison.

UNet 3+ is built upon UNet++, just as UNet++ is built upon U-Net (Huang H. et al., 2020). According to the authors, there is still a lot of space for development in UNet++ because it does not sufficiently investigate data from full scales. Learning the position and boundary of items explicitly is necessary in order to explore the entire scope of the input. This is particularly helpful for medical imaging, which may show organs of various sizes. They aim to decrease the number of parameters needed while also improving performance and accuracy. Once more, the skip connections are where the change occurs. In contrast to UNet++’s thick and hierarchical skip connections, UNet 3+ employs full-scale skip connections. Figure 4 shows how various architectures and their skip connections may be compared. As a result, UNet 3+ can use the dice metric to obtain more accuracy with fewer input parameters. However, a dataset that is better appropriate for the UNet 3+ architecture is used to test accuracy. Given the image, the dataset contains livers and spleens of various sizes.

#### 4.2.4 NAS-UNet

Artificial neural networks can be generated automatically using NAS (Neural Architecture Search). For hyperparameter optimization, NAS can be employed in a manner akin to evolutionary algorithms. A search space, a search approach, and a performance estimation approach make up NAS. NAS algorithms can be broadly divided into three categories: evolutionarily computation (EC)-based NAS algorithms, gradient-based NAS algorithms, and RL-based NAS algorithms (ENAS) (Liu et al., 2020). Weng et al. (2019) utilized NAS for the first time to segment medical images. The core of the NAS-Unet model is the UNet architecture, and NAS is used to identify an optimal design that performs better than other UNet versions for 3D semantic segmentation. The authors also dramatically lowered the number of parameters when compared to UNet. The fundamental principle of NAS-Unet is to develop two cell architectures, DownSC and UpSC, and then use NAS to identify the most optimized versions of these cells. Data in the encoder portion of the UNet is downscaled using a block called DownSC, whereas data in the decoder portion of the UNet is upscaled using a block called UpSC. They choose a set of basic operations for the cells that call for their knowledge. According to the authors, the most well-liked and effective CNN architecture for image classification is used to select the procedures. They also value procedures with fewer parameters and no redundancy. While the latter aims to lower the number of parameters compared to the original UNet, the former refers to each operation having a few distinctive qualities. They used DARTS (Liu et al., 2018), a gradient-based NAS algorithm, as inspiration for their search technique. Adopting Binary Gate, which updates only one architecture parameter by gradient descent at each step, the authors modify DARTS to quicken the search process. Unlike DARTS, which performs step-by-step modifications to all architecture parameters, the Promise12, Chaos and NERVE datasets are subjected to NAS-Unet. The datasets include medical images produced by ultrasound, computed tomography (CT), and magnetic resonance imaging (MRI) (Weng et al., 2019). The authors come to the conclusion that NAS-Unet performs better than standard techniques like U-Net and FC-Densenet while also requiring less parameters.

### 4.3 Voting mechanism

In our system, multiple learning algorithms are used to achieve better segmentation performance. To find the global segmentation results, ensemble learning via a voting mechanism is used. A common voting method in ensemble segmentation is majority-based voting. Another name for it is plurality voting. The method presented here uses a majority-based voting mechanism to improve the segmentation results after using the four segmentation algorithms described above. For each test instance, the results of each model segmentation are calculated and the final output is determined based on the results that are in the majority. By using majority voting (plurality), each model predicts the class label for each pixel in the output image in majority voting.

### 4.4 Hyper-parameter optimization

It is necessary to determine the optimal learning rate, epoch number, and batch size for the designed deep learning architectures. Each of these hyperparameters directly affects the performance of the model without changing the architecture. We optimize the hyperparameters using evolutionary techniques. A population of individuals consisting of genes forms the genetic algorithms (GAs). The learning rate, the number of epochs and the batch size of the four architectures (U-Net, UNet++, UNet 3+, and NAS-UNet) are the hyperparameters considered in this work. Then, the individuals in the environment are evaluated. When the model is run with their genes, or hyperparameters, it results in a loss. Then, an individual’s fitness is determined by their loss; a bigger loss results in a lesser fitness, and vice versa. This generation is finished once all members of the population have undergone fitness tests and gotten their results. The last step is to figure out which individuals will be part of the generation. In order to achieve this, parents are chosen based on their ability to reproduce; greater fitness increases the likelihood of becoming a parent; survival of the fittest. The parents then replenish the population of the following generation, and the cycle repeats. The GA will ultimately converge to a global optimum where the majority of individuals contain the ideal model hyperparameter values.

**FIGURE 5:**
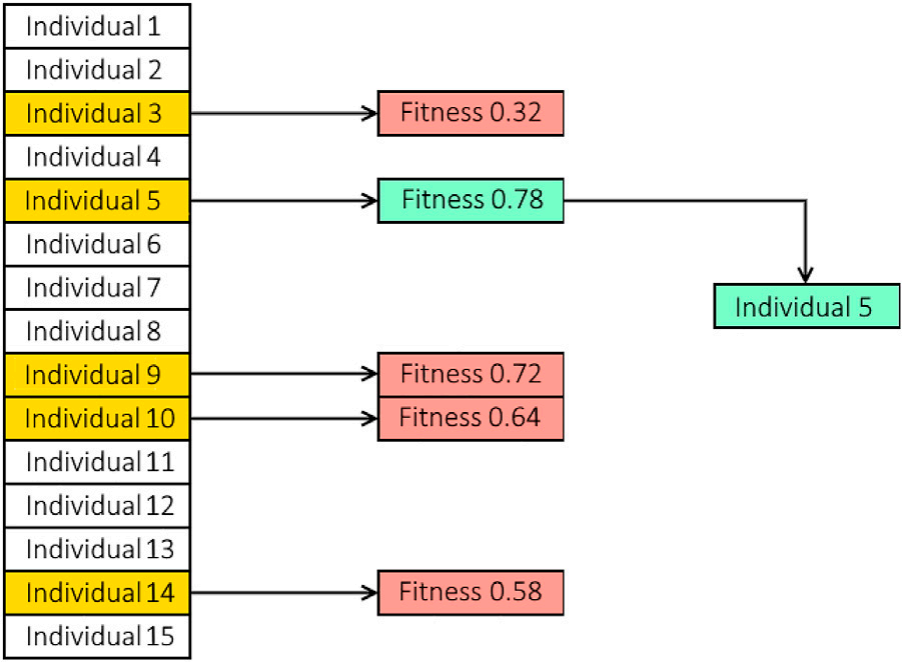
A population containing 15 individuals where tournament selection is applied. At random, five individuals are randomly chosen to compete in the tournament. The winner of the tournament is based on the predetermined fitness of the individual. In this case, individual five wins the tournament and is selected to be a parent.

• Defining the search space: the learning rate, the number of epochs, and the batch size have been specified as the components of the GA’s search space. We still need to specify and explain the scope of the hyperparameters, though. Depending on the restrictions placed by the hyperparameter itself, the range may be quite arbitrary. For instance, the learning rate could only be set to between 0 and 1. It is somewhat wasteful to add such a high learning rate even when it is never utilized.
• Selection: Additionally, we need to choose the individual who will make up the future generation. We applied a tournament-based strategy, which randomly chooses a group of individuals from the population. The winner of the “tournament” is chosen to be a parent after the set competes. The winner of the competition will be the one with the highest level of fitness, therefore the outcome is somewhat pre-determined. The competitors in the competition are not subjected to any new tests. The selection process for tournaments includes some built-in exclusions for the very lowest performers. Let us say there are 100 individuals in the population and five individuals are competing. The four individuals who perform the worst can never be chosen as parents given these values. There is no tournament set in the populace where these four can prevail. Figure 5 illustrates a selection tournament example that is applied to the suggested framework.
• Crossover: The chosen parents must reproduce and give creation to new individuals after submitting an application for tournament selection and locating the set of parents. The new individual is made up of a combination of the DNA from the parents. We can transform the values to binary representations or use the genes as their values while executing crossover. Successful parents receive the crossover process, but it has the potential to alter the order of the many genes. Every possible gene combination is included in the search space, and the crossover process aids in the systematic exploration of the various combinations. Utilizing a binary format allows for even deeper investigation of the crossover process because it gives each gene’s value the opportunity to be changed.
• Mutation: There is a possibility that a newly produced individual will become mutated. The individual’s genes are changed via mutation. By changing the gene’s value, mutation might occur to one gene or many genes. Within the limitations of the gene, alternation causes the gene to be randomized. New genes are added to the population through mutation. The modified individual bearing the new genes will be quickly wiped out of the population if these genes are bad, that is, if they score a poor fitness. If the genes are sound, however, the GA will continue to use the newly discovered genes and procreate the population.

### 4.5 Blockchain technology

In this section, we will describe our blockchain-based strategy used in BIoMT-ISeg. Every block in a blockchain is connected to its predecessor through a backward reference, which is the hash value of the predecessor block. For instance, block *i + 1* contains the hash value of its previous block *i*. Each block also contains other data fields, such as the input data for training, and the hyperparameters of the trained model. The process starts by having the data (input/output data, model architecture, weights, and model parameters) in the form of a transaction, a device sends a transaction to the blockchain using *SHA*-*512*, in 64-bits words. The length of the hashed data is extended to 1,024 bits or a multiple of that number. The message is then split into blocks of 1,024 bits and tagged with an *S*_1_, *S*_2_,…, *S*_*n*_ in the following stage, where *n* is the number of all sites in the Internet of Things (IoT) network. Blocks are produced in this manner, and since the initial hash value is known and designated as $H^{0}$ , the following action can be recursively carried out for each site *S*_*i*_ using Eq. 1:

$$H^{i}=H^{i-1}+C\left(H^{i-1}\right)\%64$$ (1)

Note that the functions “C”, and “%” denote the compression, and the modulo functions, respectively.

In our blockchain strategy, we also used smart contracts, which automate the execution of contractual terms and clauses when specific conditions are met and can run on top of blockchain. Smart contracts are immutable due to the immutability of blockchains and the root hash of all the smart contracts after being compiled into bytecode and stored in blockchains. Smart contracts can reduce potential hazards, improve business activity efficiency, and simplify administration. Each site looks over the smart contract and decides which entities could create additional blocks. The contract is also sent electronically to the sites. The different blocks that make up each site contain a cached copy of the smart contracts. The automatic sharing of sensitive information with other websites is then possible using these barriers. Request for authentication will be granted by each site in order to share data. With the certificate authority, each website must first sign a registration agreement before storing both its public and private keys in a safe place. To keep the data’s confidentially intact while being sent across the various sites that make up the proposed system, an encryption system must be used. The certification authority has continuously verified both the data source and the destination. The transmission is terminated and a report is generated blacklisting the IP address of the discovered site if the data is sent to or received from an invalid site. After the certification authority has verified the validity of the transfer, the signature and encrypted data are both sent back to the specified site. Then, a saved request from the specified website containing this information is delivered to the blockchain system.

## 5 Performance evaluation

Intensive simulation of the proposed solutions on a federated learning environment uses IoMT setting in several medical image datasets. The detailed description of these datsets is given in the following:

1) Ultrasound nerve segmentation^1^ **:** The dataset contains 5,600 images. The goal is to segment a group of nerves known as the brachial plexus in ultrasound images.
2) Brain image segmentation^2^ **:** It offers information on four modalities (T1, T1w, T2, T2 FLAIR) of MRI images, as well as patient survival statistics and expert annotations. It is 8 GB in size with 1,484 images.
3) Breast ultrasound images dataset^3^ **:** The data examines medical images of breast cancer obtained via ultrasound scan. Breast Ultrasound Dataset images are classified into three types: normal, benign, and cancerous. Breast ultrasound images were gathered at the start of the study from 600 women aged 25 to 75. The dataset contains 780 images, each of which is 500*500 pixels in size.
4) COVID-19 radiography database^4^ **:** It includes chest X-ray images for COVID-19 positive cases, as well as photos of normal and viral pneumonia. It includes 3,616 COVID-19 positive cases, 10,192 Normal, 6,012 Lung Opacity (Non-COVID lung infection), and 1,345 Viral Pneumonia images and lung masks.

The evaluation of the proposed framework is calculated using the following Intersection-over-Union (*IoU*), (Eq. 2) among the ground truth *U*, and the model output *V*:

$$IoU\left(U,V\right)=\frac{\left|U\cap V\right|}{\left|U\cup V\right|}$$ (2)

IoU is a method of measuring the overlapping labels. It measures the overlapping true and false labels, then divides it by the union of the labels, the pseudo code of the algorithm is given in Appendix 1.

### 5.1 Parameter setting

**FIGURE 6:**
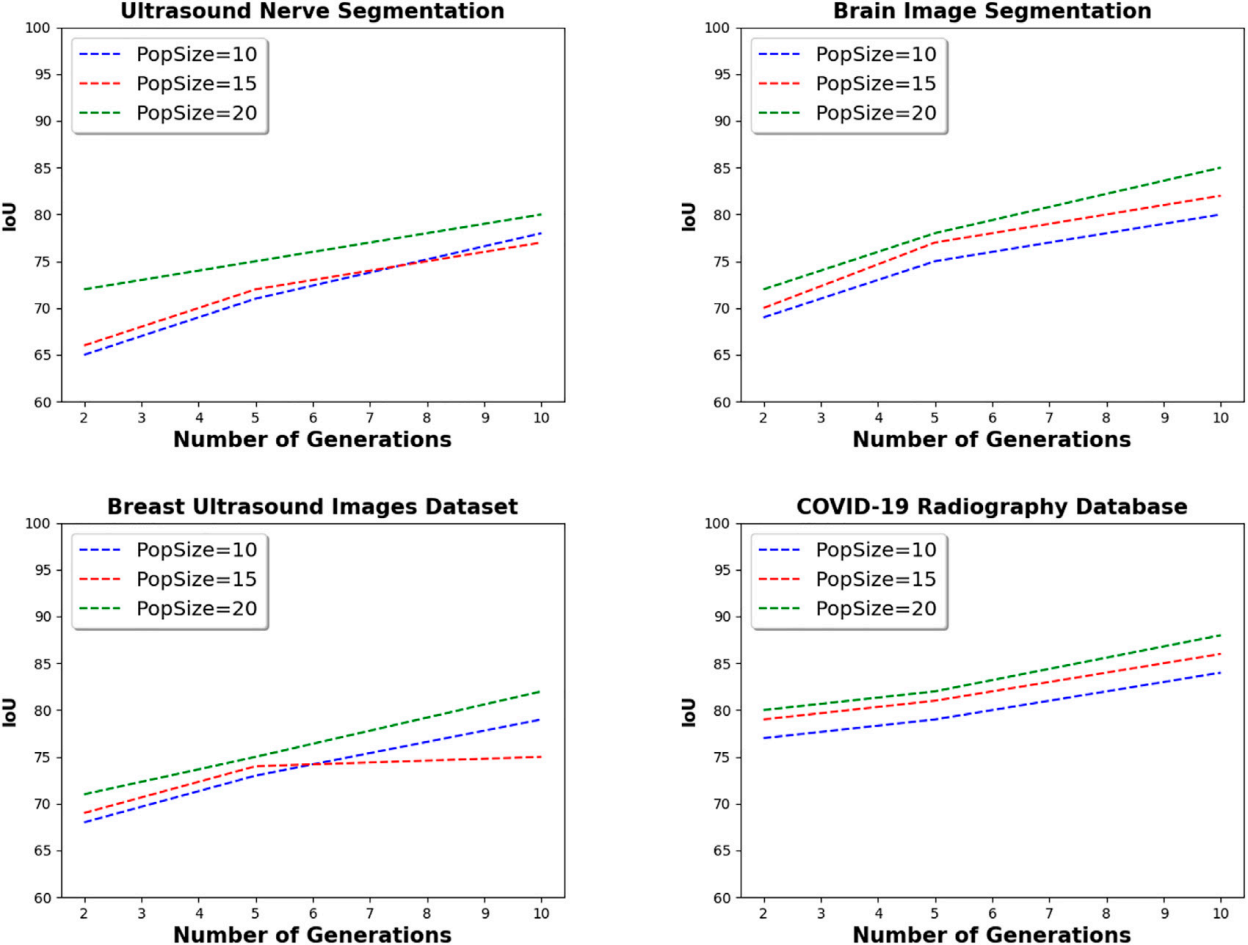
Parameter settings of the proposed framework using medical image datasets.

Figure 6 presents the parameter setting step of the proposed framework. By varying the number of generations from 2 to 10, and the population size from 10 to 20, the IoU value of the proposed framework is increased to whatever the datasets used in the experiments. For instance, the IoU value is only 69 with two generations, and 10 individuals on Brain Image Segmentation dataset. However, with the same dataset, and with 10 generations, and 20 individuals, the IoU value is 85. These results can be explained by the fact that with more generations, and more individuals, the developed evolutionary strategy explores more configurations and therefore retrieves high quality models. In the remainder of the experiments, the number of generations is set to 10, and the population size is set to 20.

### 5.2 Quality of segmentation performance

**FIGURE 7:**
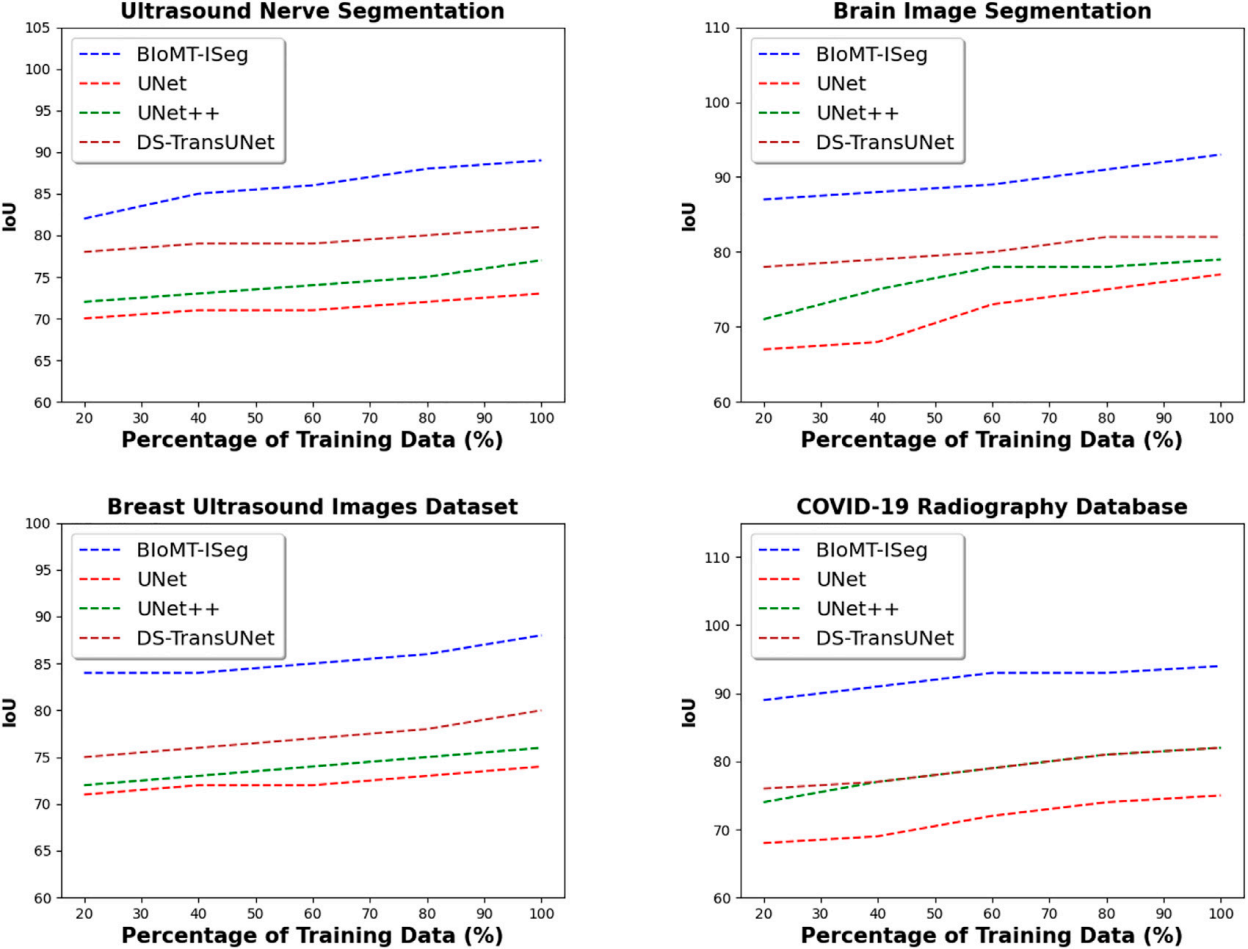
IoU of the proposed framework, and the baseline models using medical image datasets.

For the remainder of the experiments, we divided the data into training and testing data. From the training data, we divided it into several buckets. The first bucket contains 20% of the training data, the second 40% of the training data, and so on until the last bucket contains 100% of the training data. Figure 7 presents the IoU of the proposed solution, and the baseline methods on the four datasets used in the experiment. By varying the percentage of training images from 20% to 100%, the results indicate that BIoMT-ISeg outperforms the three other algorithms in terms of IoU. Indeed, the IoU of BIoMT-ISeg goes up 94%, where the IoU of the baseline solutions does not exceed 82%. These results are obtained thanks to the hybrid segmentation model used in this research work, where several segmentation models have been incorporated in order to achieve the best segmentation results. In addition, determining the best configuration of the model using the genetic algorithm gives better quality of the segmentation output.

### 5.3 Blockchain performance

In the final experiment, the suggested framework will be contrasted with and without the use of blockchain technology. Table 1 shows the number of detected attacks with and without using the blockchain technology. The outcomes clearly demonstrate the suggested framework’s advantages when leveraging the blockchain. The results gained support our argument that by examining the blockchain paradigm, we may overcome the security issue with medical applications.

**TABLE 1:** Percentage of detected attacks with and without using the blockchain technology.

Dataset	Percentage of data	With blockchain	Without blockchain
Ultrasound Nerve Segmentation	10	76	15
	20	75	14
	40	71	12
	60	70	13
	80	71	11
	100	73	12
Brain Image Segmentation	10	76	15
	20	75	14
	40	71	12
	60	70	13
	80	71	11
	100	73	11
Breast Ultrasound Image Dataset	10	76	15
	20	75	14
	40	71	12
	60	70	13
	80	71	11
	100	73	11
COVID-19 Radiography Database	10	76	15
	20	75	14
	40	71	12
	60	70	13
	80	71	11
	100	73	11

### 5.4 Visualization

**FIGURE 8:**
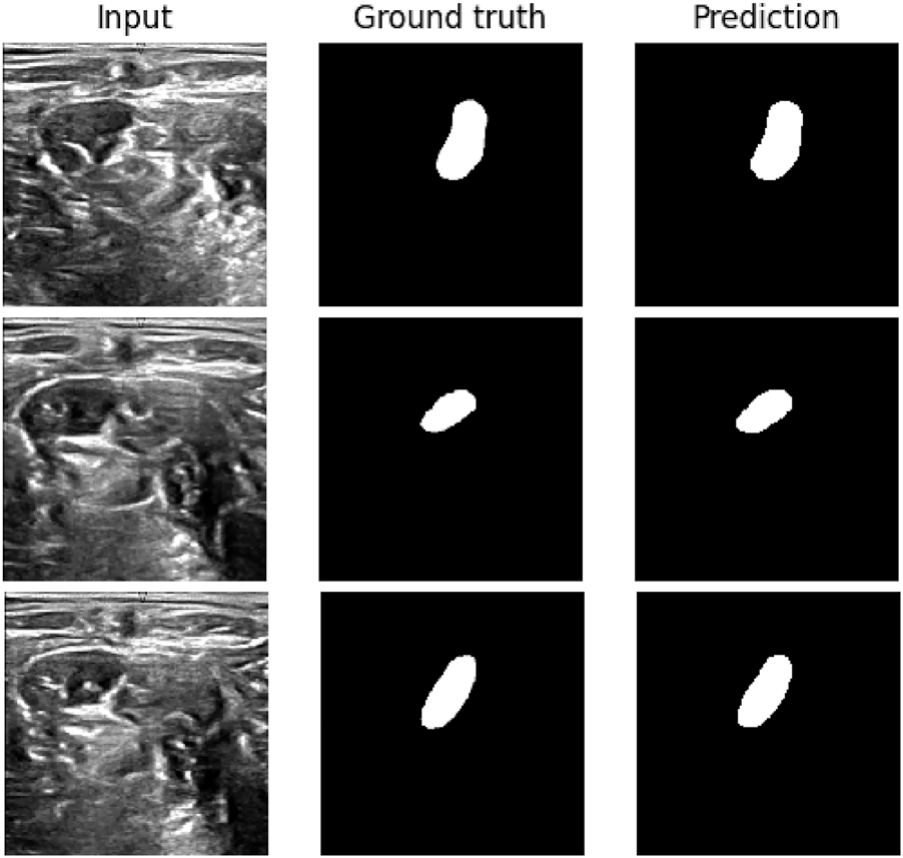
Ultrasound images of the neck as input on the left side, center shows the hand annotated nerve, right side shows the prediction by the model. These are some of the best results.

Figure 8 shows three images: the input, the manually labeled neuron, and the segmentation prediction. These results show a clear convergence between the results of the created method and the actual data. Moreover, the proposed approach can detect different shapes and sizes, which is quite difficult for the most advanced algorithms. These results are made possible by the segmentation technique, which employs effective hyperparameter optimization to find the ideal parameters for the proposed model.

## 6 Discussion and future direction

IoMT has shown impressive results for various medical applications, but medical image segmentation has attracted a lot of research, since many sensitive medical scenarios need this task. For instance, brain tumor detection needs to segment MRI images in order to identify the location of the different tumors in the brain. BIoMT can be utilized in some specialized situations where data can not be secured by the traditional internet protocols. The designs of such system can be improved from a variety of perspectives due to their stage of development. High accuracy, high privacy, and low cost are all desirable design outcomes. Although there has been a lot of interest in the development of IoMT in recent years, only a few solutions are capable of being applied in real-world medical settings. We will discuss next our perspectives on the basic difficulties and technological shortcomings faced by IoMT to improve BIoMT-ISeg, which are crucial to their successful development toward medical applications and ubiquitous medicine:

1) Interpretation: Deep learning has recently made a lot of progress, and the success of artificial intelligence (AI) in the medical industry has resulted in a significant surge in IoMT applications. IoMT research aims to develop applications that leverage AI technologies to assist clinicians in making medical decisions (Mishra et al., 2022). IoMT is used for solving various medical problems such as disease diagnosis (Verma et al., 2022), surgery (Han et al., 2022), and many others. However, IoMT applications encounter various problems, such as the black-box nature of deep learning models. Because these black-box models are difficult to explain, medical specialists are hesitant to make explainable clinical judgments. Deep learning models frequently have millions of parameters and simply return a final decision result with no explanation. Because deep neural networks lack transparency, it is difficult for the user to determine whether the choice is reliable, jeopardizing trust with doctors. IoMT must be transparent in order to gain the trust of doctors. Explainable artificial intelligence (XAI) research has recently received a lot of interest (Djenouri et al., 2022). For medical AI applications to be accepted and integrated into practice, XAI is crucial. As future perspective, we plan to integrate XAI solutions into the developed BIoMT-ISeg framework.
2) Neural Evolving: The manual fine-tuning of existing networks to apply on a new application and/or dataset is a traditional way for designing a new network Lu et al. (2022). In most circumstances, trial and error is a time-consuming method that is frequently utilized to find a viable solution. The issue is the variety of parameters that must be configured in order to reconstruct a network for a new application. Furthermore, in most circumstances, the available data and computer resources are restricted, making this work considerably more difficult. More crucially, most created networks are only effective on a single application or dataset. As a result, they must be updated in order to be used in a new application. Neuroevolution is a technology that uses evolutionary computation to determine the architecture and parameters of a neural network (Huang et al., 2022; Kyriakides and Margaritis 2022). As a future perspective, we plan to integrate neuro-evolving strategy into the developed BIoMT-ISeg framework.

## 7 Conclusion

Although U-Net based models have excellent behavior for handling segmentation problems in medical applications, numerous constraints remain unresolved. In this paper, we provided solutions for the learning process, the hyperparameter optimization and privacy challenges by establishing an end-to-end intelligent framework that combines ensemble learning, the genetic algorithm, blockchain technology, and the different U-Net based architectures to obtain the best accuracy in training complex medical data for IoMT situations. The learning rate, batch size, and number of epochs hyperparameters were optimized using genetic algorithms. Blockchain technology was also used to safeguard the training process. Ensemble learning was finally established to merge the local outputs of the different segmentation models to the global one using a voting mechanism. Our technique demonstrates that a high learning rate and a small batch size can result in good performance in a small number of epochs. This enables us to outperform baseline solutions for well-known medical databases.

## Data Availability

The original contributions presented in the study are included in the article/Supplementary Material, further inquiries can be directed to the corresponding author.

## Footnotes

1
https://www.kaggle.com/competitions/ultrasound-nerve-segmentation/data

2
https://www.kaggle.com/datasets/andrewmvd/brain-tumor-segmentation-in-mri-brats-2015

3
https://www.kaggle.com/datasets/aryashah2k/breast-ultrasound-images-dataset

4
https://www.kaggle.com/datasets/tawsifurrahman/covid19-radiography-database

## Appendix

This section contains some of the core code snippets used to execute our solution. The code snippets contains abstract methods, but the purpose of the methods should be evident.

The main loop which iterates through the selected number of generations.
